# The role of disorder in RNA binding affinity and specificity

**DOI:** 10.1098/rsob.200328

**Published:** 2020-12-23

**Authors:** Diana S. M. Ottoz, Luke E. Berchowitz

**Affiliations:** 1 Department of Genetics and Development, Columbia University Irving Medical Center New York, NY 10032, USA; 2 Taub Institute for Research on Alzheimer's and the Aging Brain, Columbia University Irving Medical Center New York, NY 10032, USA

**Keywords:** RNA-binding proteins, RNA-binding modules, RNA-binding domains, intrinsically disordered regions, linkers, assemblies, amyloids

## Abstract

Most RNA-binding modules are small and bind few nucleotides. RNA-binding proteins typically attain the physiological specificity and affinity for their RNA targets by combining several RNA-binding modules. Here, we review how disordered linkers connecting RNA-binding modules govern the specificity and affinity of RNA–protein interactions by regulating the effective concentration of these modules and their relative orientation. RNA-binding proteins also often contain extended intrinsically disordered regions that mediate protein–protein and RNA–protein interactions with multiple partners. We discuss how these regions can connect proteins and RNA resulting in heterogeneous higher-order assemblies such as membrane-less compartments and amyloid-like structures that have the characteristics of multi-modular entities. The assembled state generates additional RNA-binding specificity and affinity properties that contribute to further the function of RNA-binding proteins within the cellular environment.

## 1. Introduction

**Figure 1.**
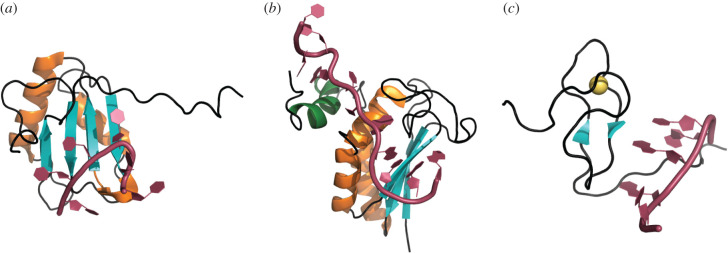
Selected examples of RNA-binding modules. (*a*) RRM1 of the polypyrimidine tract-binding protein (PTB) interacting with CUCUCU RNA (PDB ID 2ad9) [3]. (*b*) KH domain and QUA2 region of splicing factor 1 (SF1) complexed with UAUACUAACAA RNA (PDB ID 1k1g) [4]. (*c*) Zinc finger of FUS interacting with UGGUG RNA (PDB ID 6g99) [5]. In all panels, RNA is represented in magenta. Protein α-helices are depicted in orange (with the exception of the QUA2 region, which is depicted in green), β-sheets in teal and loops in black. The zinc atom in (*c*) is depicted as a yellow sphere. Structures were visualized with PyMOL, version 2.4.

All steps of metabolism and function of a large fraction of RNAs, including messenger (m) RNAs, ribosomal (r) RNAs and several non-coding RNAs, require interaction with proteins. RNA–protein complexes are termed ribonucleoprotein (RNPs) particles [1,2]. RNA-binding proteins (RBPs) have a typical modular structure, where RNA-binding, catalytic and regulatory elements are combined together to define the targets and the function of these proteins [2]. In this review, we refer to discrete structural elements that are able to carry out an independent function as modules. RBPs contact RNA through structural elements called RNA-binding modules (figure 1). Individual RNA-binding modules typically bind between two and six nucleotides, underscoring their generally low binding affinity and sequence specificity [2,6,7]. Nevertheless, many RBPs have specific targets and can bind them tightly, raising the question of how these proteins reach the binding abilities necessary for their function. To achieve proper affinity and specificity, most RBPs combine several RNA-binding modules through disordered linkers [2,8,9]. Furthermore, RBPs typically contain extended intrinsically disordered regions (IDRs) that have the ability to mediate protein–protein and RNA–protein interactions [10,11]. These flexible regions facilitate the formation of large non-stoichiometric assemblies, such as membrane-less compartments and amyloid-like structures [12–14]. The highly varied functions of these assemblies reflect a diverse composition, raising the question of how selected proteins and RNAs are recruited to the correct assembly.

In this review, we discuss the modular characteristics of RBPs and how modularity is exploited to mediate interactions with the target RNA. We illustrate the crucial role of IDRs in defining RNA-binding affinity and sequence specificity. IDRs often behave as linkers connecting RNA-binding modules within a single RBP where they dictate how modules are arranged and relate to each other. The structural constraints and topologies defined by linkers define the RNA targets of a single RBP. IDRs can also function to connect structural elements between and among proteins and RNA promoting the formation of higher-order RNA–protein assemblies. We propose an analogy between intra-protein linkers and assembly-promoting IDRs. As in individual proteins where IDRs link RNA-binding modules, in higher-order assemblies, IDRs can connect proteins and RNA resulting in complexes that have the characteristics of multi-modular entities. The assembled state can result in new RNA-binding specificity and/or affinity that further the function of RBPs within the cell.

## 2. RNA-binding modules are versatile elements with variable affinity and specificity for RNA

Before we discuss how RNA-binding modules combine with one another, we first briefly review the principles underlying their characteristics. RNA-binding modules are versatile elements (figure 1). Although some bind both RNA and DNA, specificity for RNA is usually obtained through recognition of unique RNA chemical moieties such as the 2′ OH on the ribose, or secondary structures [7,15]. RNA-binding modules can bind single-stranded or double-stranded RNA [2,7,8]. Depending on which parts of the nucleotides are contacted (i.e. bases or backbone phosphate and sugar), RNA-binding modules may have sequence specificity [7,8]. Many of these modules have been identified and characterized. For a detailed discussion of the structural and biochemical information available for RNA-binding modules, we address the reader to other reviews [2,7,8,16].

The RNA-recognition motif (RRM) is a common and well-characterized RNA-binding module and here we use it as an example of how an element recognizes and binds RNA [2,8]. The RRM contains 80–90 residues and contacts two to eight nucleotides [17]. Its topology consists of two α-helices packed against an antiparallel β-sheet (figure 1*a*). Within the β-sheet, two conserved motifs, RNP1 and RNP2, contact two contiguous nucleotides, conferring specificity for single-stranded RNA [17,18]. RNP1 and RNP2 can bind several combinations of dinucleotides, demonstrating that RRMs can in principle accommodate different targets on their β-sheet. Nevertheless, some RRMs are known to bind specifically and/or with high affinity [18]. This is achieved through additional contacts mediated by various residues on the β-sheet surface and at the N- and C-termini of the module [18]. Variations of the fundamental structure also contribute to binding affinity and specificity. The length of the secondary structure elements and the loops connecting them can vary, providing additional contacts with RNA [17,18]. These additional contacts allow for target discrimination by sequence and, in some cases, by shape [19]. For example, the RRM of the human splicing factor hRBMY exhibits binding specificity to a synthetic pentaloop capping a stem *in vitro*. Target recognition involves the β-sheet binding a triplet within the pentaloop and the *β*2/*β*3 loop identifying the RNA stem through its shape [20]. An extreme case illustrating the versatility of the RRM is given by hnRNP F, which is involved in post-transcriptional RNA processing. This protein contains three ‘quasi-RRMs’ with poorly conserved RNP1 and RNP2 motifs [21,22]. These quasi-RRMs contact poly(G) sequences specifically through residues placed within the loops connecting their secondary structure elements and do not use the β-sheet for binding [21]. Extensions of the RNA-binding surface to increase sequence specificity and/or binding affinity have been observed in RNA-binding modules other than the RRM, such as the K homology (KH) domain [8] (figure 1*b*). This module contains approximately 70 residues organized in an antiparallel β-sheet packed against three α-helices. These structural elements, together with other elements contained in the *α*1/*α*2 and *β*2/*β*3 loops, form a binding cleft contacting four single-stranded nucleotides with low affinity [23]. Extensions of the KH domain increase binding affinity and/or specificity. For example, the KH domain of the splicing factor SF1 contains a C-terminal conserved region called Quaking homology 2 (QUA2) that forms an α-helix that extends the KH-binding surface. This extension enables the formation of a large hydrophobic groove that specifically binds ACUAAC found within the intron branchpoint sequence UACUAAC [4]. The above examples illustrate how structural variants within a single RNA-binding module can provide a vast range of sequence specificity and binding affinity. Beyond their fundamental sequence and structural features, these structures exhibit conformational flexibility that further contributes to binding affinity and specificity.

The intricate contacts established between RNA and proteins often cannot be explained with a rigid lock-and-key docking model [15,24]. Comparisons of free and bound structures show that RNA-binding modules and RNA undergo conformational changes when they interact with each other [15]. In most cases, these changes consist of the local reorganization of loops connecting secondary structure elements for the RNA-binding module and local changes in tertiary or secondary structural elements for the RNA [8,15,18]. The conformational changes associated with RNA–protein interactions are possible because most of the interfaces of proteins and RNA do not have a unique and stable conformation, but exist as several energetically favourable conformers [15]. The conformational dynamics of interacting interfaces can be rationalized with two opposing mechanisms. Induced fit, first formulated to describe how some enzymes are activated for catalysis, describes conformational changes that occur as a consequence of the interaction [24]. Conversely, the conformational selection mechanism describes how the ligand selects an existing free conformation of the binder [9,15]. The conformational flexibility of free RNA and proteins influences binding specificity and affinity. Rigid interfaces usually favour specificity because they provide inflexible docking for the ligand. A certain level of flexibility increases binding affinity, because it enables the optimal juxtaposition of the interacting interfaces [15]. Modifying enzymes can alter the flexibility of RNA and protein surfaces, resulting in potent regulation of RNA and protein interactions [25–28]. Overall, affinity and specificity of individual RNA–protein interactions depend on the structural features of both RNA and RNA-binding modules and on the level of conformational flexibility of these interacting partners.

## 3. Disordered linkers combine and coordinate multiple RNA-binding modules within a protein

**Figure 2.**
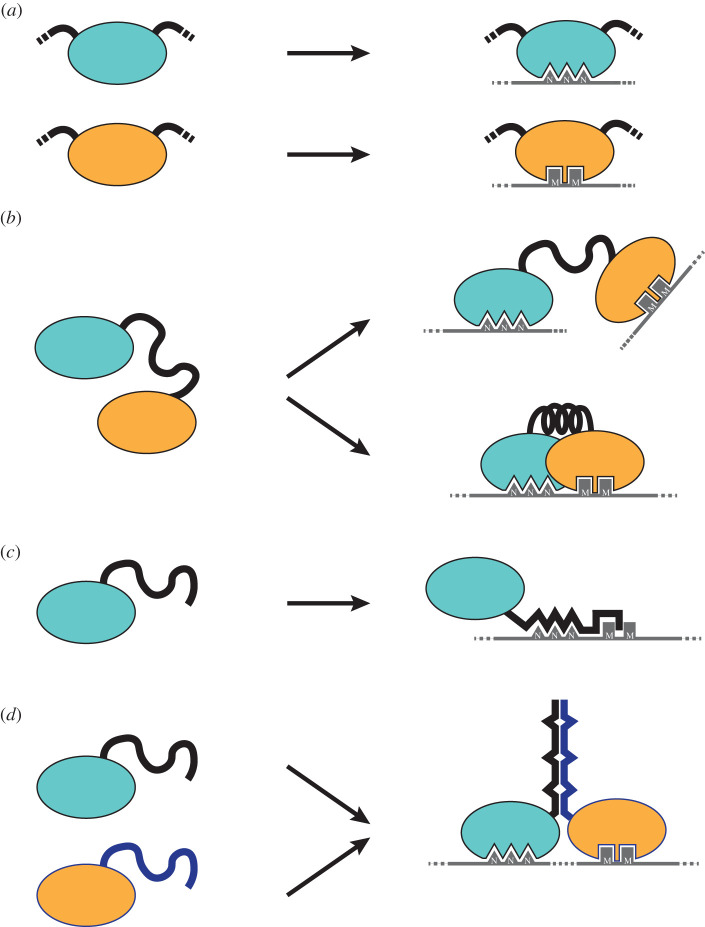
RNA-binding proteins have a modular structure where intrinsically disordered regions (IDRs) can work as linkers connecting modules within a single protein or as connectors that mediate interactions between several proteins and/or RNA. (*a*) RNA-binding modules typically recognize short sequences on the RNA. (*b*) Linkers connecting RNA-binding modules are usually disordered in the free state. Some linkers stay disordered upon RNA binding and enable the RNA-binding modules to contact their RNA targets independently (top). Linkers can mediate molecular recognition by undergoing a disorder-to-order transition in the RNA-bound state and enable cooperative binding (bottom). (*c*,*d*) IDRs can interact with RNA (*c*) and proteins (*d*), promoting the formation of multimeric complexes. NNN and MM represent two different RNA sequence motifs.

Individual RNA-binding modules typically recognize short RNA stretches and bind them with low affinity [2,7]. To achieve sequence specificity and higher binding affinity, RBPs combine several RNA-binding modules (figure 2*a,b*). These combinations collectively define the RNA-binding properties of the protein [2,9]. For example, the polypyrimidine tract binding protein (PTB) involved in post-transcriptional RNA processing and translation uses four RRMs to bind poly(CU) tracts [3]. By contrast, FUS contacts RNA in several contexts of RNA metabolism through a combination of different types of modules, such as an RRM, a zinc finger and several RGG repeats [5] (figure 1*c*).

The linkers connecting RNA-binding modules play a crucial role in RNA–protein interactions because they control how these modules can move relative to each other, defining their spatial separation, their orientational freedom and their effective concentration [2,9,29,30]. Linkers are usually intrinsically disordered, that is, they do not fold autonomously into a single well-defined tridimensional structure. Instead, these IDRs sample a distribution of conformations called an ensemble [31–33]. The inability of IDRs to fold into a single well-defined conformation is generally a consequence of their enrichment in polar and charged residues and their lack of bulky hydrophobic amino acids that form the well-structured hydrophobic core of globular domains [34,35].

While some disordered linkers connecting RNA-binding modules do not contact the modules or the RNA, others do establish these contacts [9] (figure 2*b*). Linkers that do not establish direct contacts with other proteins or nucleic acids in the bound state, but remain unstructured, confer a dynamic disorder connotation to the complexes [9,36]. These linkers allow RNA-binding modules to bind to non-contiguous binding sites within the same RNA or on different molecules [2,9]. This is the case for the linkers connecting the first, second and third RRM of PTB [3] and the three quasi-RRMs of hnRNP F [21]. These linkers increase the chance of interaction with RNA because they enable the RNA-binding modules to contact their targets independently [8]. Linker length and flexibility control the maximal distance possible between two RNA-binding modules and define the volume that a module samples once the other is bound [2,6,9]. Therefore, the linker controls the local concentration of the free module and hence influences its binding affinity. If the linker is infinitely long and flexible, the two RNA-binding modules can be considered as independent entities, and the total binding affinity is the sum of each. Shorter linkers increase binding affinity because they increase the effective local concentration of the free module. To the extreme, the total binding affinity of two modules rigidly connected by a linker or interacting with one another is expected to be the product of the two [37].

Disordered linkers that enable molecular recognition by establishing contacts with the RNA and the RNA-binding modules allow for cooperative RNA binding [8,18] and undergo a disorder-to-order transition upon interaction [29,30,38,39]. This conformational transition can involve the entire IDR or short internal sequences [31,38,39]. Similar to the principles underlying the interactions between proteins and RNA, two opposing mechanisms have been proposed to rationalize disorder-to-order transitions. The induced folding mechanism describes how the IDR first associates with its binding partner and then undergoes folding. The conformational selection mechanism describes how the binding partner selects a conformation of the IDR among those sampled in its free ensemble [39]. Several examples of linkers that undergo a disorder-to-order transition have been characterized, including those connecting the two RRMs found in Sex-lethal [40] and Hrp1 [41], involved in alternative splicing and in precursor messenger (pre-m) RNA 3′ end processing, respectively. In their free state, these RRMs tumble independently, as the linker connecting them is disordered [41,42]. When RNA is present, the linker becomes partially structured and assists the rearrangement of the two RRMs. The RRMs, together with the linker, form a large RNA-binding surface and the linker directly interacts with RNA [40,41]. This binding architecture has also been observed in HuD, another post-transcriptional regulator. Remarkably, the RNA sequences recognized by Sex-lethal, Hrp1 and HuD are very different, exemplifying the binding versatility of the system [17,41]. These examples illustrate how RNA–protein interactions depend on the juxtaposition and interactions between RNA-binding modules that are controlled by the linkers connecting them. These features together finely control target specificity and binding affinity of an RBP.

## 4. Intrinsically disordered regions can bind RNA independently of their role as linkers between RNA-binding modules

RNA-binding IDRs are widespread in the proteome and can be found either alone or associated with structured RNA-binding modules [25,43] (figure 2*c*). RNA-binding IDRs exhibit distinguishing features. They are usually enriched in residues that are typically found in structured RNA-binding modules such as arginine, lysine, histidine and the order-promoting aromatic residues tyrosine and phenylalanine [25,43,44]. Moreover, these IDRs frequently consist of sequences of low complexity [43], which can consist of perfect repeats of a single amino acid generally shorter than twenty residues or a highly repetitive region of more than 100 residues containing few different amino acids [45]. Arginine-rich motifs (ARMs) are stretches of 10 to 20 residues that bind their target mostly specifically and with high affinity [46]. These sequences are frequent in viral proteins [27]. Arginine can also be found in combination with other residues; arginine and serine repeats are typically found in members of the SR protein family, mostly involved in splicing. These repeats can bind RNA directly in a non-specific way. Phosphorylation of serine can modulate binding by affecting the flexibility and charge of the IDR [27]. Arginine also often co-occurs with glycine, forming RGG/RG repeats separated by zero to four residue spacers. These repeats can mediate both RNA–protein and protein–protein interactions. Arginine methylation strongly influences the ability of these repeats to bind other proteins [47]. One notable example in which RGG repeats influence RNA binding is FUS, which contains three of these regions. One such RGG repeat interacts with RNA and contributes to increased binding affinity. Moreover, the binding of this motif to RNA affects the secondary structure of the target and promotes binding of another yet unknown RBP [5]. The high flexibility conferred to the IDR by glycine could contribute to the interaction with RNA by promoting the exposure of arginine residues [26]. Similarly, glycine is thought to drive the exposure of tyrosine in YGG repeats [25,26,43]. Beside arginine, lysine-rich segments are also found flanking structured RNA-binding modules. Although their function is still unclear, it is possible that these segments facilitate RNA–protein interactions by contacting the RNA backbone [25,43]. Finally, repeats combining positively and negatively charged residues have also been observed in RBPs; however, their role in RNA-binding is not clear yet [43,48].

## 5. Intrinsically disordered regions promote the formation of large multimeric complexes

A striking characteristic of RBPs is that they are enriched for IDRs longer than 30 residues [49] that promote protein–protein and RNA–protein interactions [11,27] (figure 2*c*,*d*). The typically expanded conformations of IDRs provide flexible and conformationally adaptable interfaces that facilitate ligand binding and enable contacts with multiple partners simultaneously [29,50]. IDR post-translational modifications such as phosphorylation, methylation and acetylation crucially regulate complex formation, because they can create or disrupt binding surfaces and/or modify the IDR's flexibility and ability to fold [47,51]. Disorder-to-order transitions help lower the energy required for structural rearrangements, favouring complex formation [15]. IDRs can play an important role in the formation of stoichiometric complexes containing both RNA and proteins. For example, several ribosomal proteins are entirely or partially disordered when unbound and undergo disorder-to-order transitions when they assemble into the ribosome [52]. Many ribosomal proteins have a characteristic tadpole-like shape, with a globular head located on the ribosome surface and an extended tail inserted deeply within the structure, establishing interactions with rRNA and other proteins. Some of these extensions undergo disorder-to-order transitions upon interaction with RNA and assist rRNA folding, and assembly and stabilization of the ribosome [53]. IDRs can also prevent the assembly of complexes when they are not necessary or in the wrong cellular context. For example, the tobacco mosaic virus builds rod-shaped viral particles in which the genomic RNA is coated by a helical array of coat proteins [54]. The building blocks of the viral particle are nucleating aggregates of coat proteins that cannot nucleate further into a rod unless the genomic RNA is present. Each coat subunit contains a disordered loop that prevents further assembly when RNA is absent. Interaction with RNA induces a disorder-to-order transition within the coat protein that allows the sizing of the viral particle to match the genomic RNA [54].

**Figure 3.**
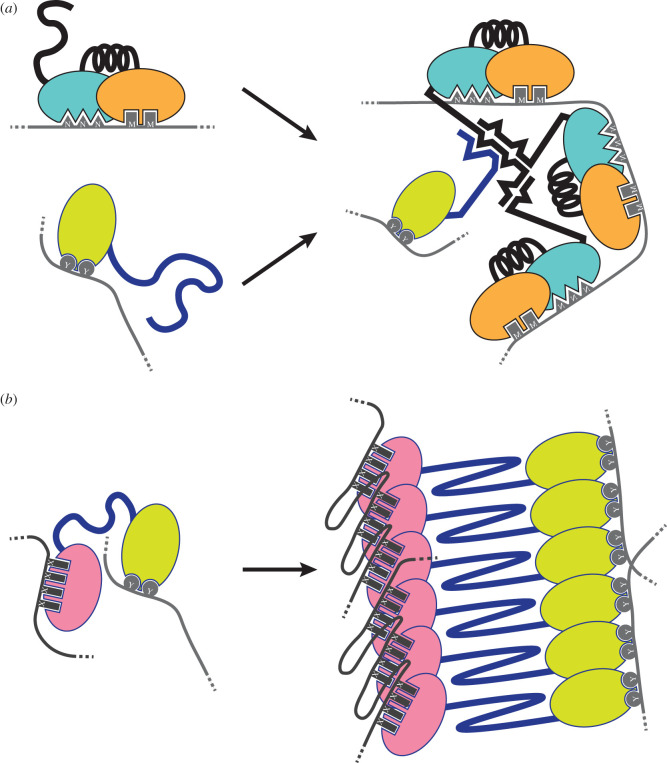
Intrinsically disordered regions (IDRs) drive the formation of higher-order assemblies, which display different RNA-binding specificity and affinity properties compared to the single components. (*a*) In membrane-less compartments, IDRs establish homotypic (depicted in black) or heterotypic (in blue) multivalent interactions, enabling the formation of multi-modular complexes of RNA-binding proteins that are able to recruit selected RNA species. (*b*) Some RNA-binding proteins assemble amyloid-like structures that can constrain the protruding RNA-binding modules to specific topologies, hence selecting specific RNA targets. NNN, MM, XXXX and YY represent four different RNA sequence motifs.

Some IDRs have the remarkable capability to drive the formation of higher-order assemblies with variable stoichiometry and heterogeneous conformation [12] (figure 3). These IDRs usually have low amino acid complexity and are often enriched in polar amino acids, such as glutamine, asparagine, tyrosine, serine and glycine [10,55]. These IDRs are sometimes referred to as prion-like domains, because they are typically contained in proteins that can form prions, a self-perpetuating templating system [10]. RBPs are particularly enriched in prion-like domains [10,56]. As much as 30% of human proteins predicted to have a prion-like domain have been annotated with the ‘RNA binding’ term in the gene ontology [57].

Low complexity IDRs can promote the formation of higher-order assemblies because they are multivalent, that is, they contain multiple binding moieties able to establish intramolecular and/or intermolecular, homotypic and/or heterotypic interactions [58,59] (figure 3). Multivalent interactions can be considered as linkers that connect modules belonging to different proteins and RNA. In a single protein, linkers covalently connect modules, affecting and integrating the functions of the individual modules [2]. Analogously, in higher-order assemblies, IDRs connect proteins and RNA through non-covalent interactions, affecting and integrating the functions of the individual molecules. These connections allow for combinations of modules not present in individual proteins that could result in the selection of new target RNAs and/or in changed RNA-binding affinities and thus new functional capabilities [6].

Within the cell, higher-order assemblies containing RBPs and RNA can form membrane-less compartments that enable the local concentration of selected proteins and nucleic acids [58] (figure 3). Several of these compartments, defined also as RNP granules, are implicated in many RNA-related processes, such as ribosome biogenesis, the transport, storage and localization of RNPs, control of translation and RNA fate, highlighting the intimate connection between these structures and RNA biology [28]. A possible but not exclusive mechanism that drives the formation of RNP granules is liquid–liquid phase separation [60,61]. This mechanism consists in the partitioning of a homogeneous polymer solution into two or more immiscible phases having well-defined interfaces [14,58,62]. Membrane-less compartments that form by liquid–liquid phase separation are referred to as biomolecular condensates [58]. Biomolecular condensates are typically multicomponent systems, resulting from the formation of networks of homotypic and heterotypic multivalent interactions [63] (figure 3*a*). These systems have a dynamic connotation [62], as they are reversible and rapidly exchange components with the surrounding environment [60,64].

Some RNP granules contain amyloid-like structures. Amyloids are highly ordered assemblies built through the repetitive addition of multiple copies of a protein or a peptide (figure 3*b*). The network formed through these homotypic multivalent interactions results in the formation of unbranched fibrils with a typical cross-β-sheet structure [65,66]. The same protein or peptide can form several distinct structures *in vivo* and *in vitro*. These fibrils may exhibit different biochemical properties and these differences could have important physiological consequences. Amyloid-like structures share a subset of amyloid-associated structural and biochemical properties such as a cross-β-sheet structure, ability to bind hydrophobic dyes and/or resistance to ionic detergents [67]. Amyloids and amyloid-like structures can form by a mechanism similar to biomolecular condensates [64,68,69] and, because of their stability, they are often considered irreversible and static [36,66]. *In vitro* phase-separated proteinaceous droplets can convert into amyloid-like structures [64,70,71]. This observation has led to the formulation of a model where biomolecular condensates and amyloid-like structures lie at the two opposite extremes of a structural *continuum* [63,64]. The ratio between heterotypic and homotypic multivalent interactions between and among the assembly components is proposed to govern the dynamics of the assembly itself; heterotypic interaction networks are proposed to buffer against homotypic interactions that drive the formation of amyloid-like structures [63]. In this model, biomolecular condensates represent a functional state, while amyloid-like structures are seen as a degenerated and therefore pathogenic state [64,70]. However, while several RBPs are functional only in dynamic assemblies, others perform their function within an amyloid-like state, serving as exceptions to this paradigm [72].

## 6. RNA-binding proteins assemble into ribonucleoprotein granules with specific identities

IDRs of RBPs are key determinants of RNP granule formation and function [14] (figure 3*a*). Deletion of the glutamine/asparagine-rich IDR of Lsm4, a multifunctional yeast RBP found in processing (P) bodies, causes loss of P bodies in some conditions. Replacement of the Lsm4 IDR with the glutamine/asparagine-rich IDR of the yeast protein Rnq1 rescues assembly of these RNP granules [73]. Similarly, the mammalian TIA-1 protein contains a glutamine-rich IDR that is required for stress granule formation. Replacement of this IDR with the glutamine/asparagine-rich prion domain of the yeast translation termination factor Sup35 recapitulates the ability of this protein to assemble stress granules [74]. These examples illustrate how IDRs can behave as connectors that promote the formation of higher-order assemblies. Moreover, these examples also illustrate the importance of IDR amino acid composition, rather than the primary sequence, in assembly formation [75].

The varied functions of RNP granules reflect a diverse composition, raising the question of how selected proteins and RNAs are correctly recruited. The weak, exchangeable, homotypic and heterotypic multivalent interactions between IDRs that drive phase separation *in vitro* play a role in defining the identity of a phase-separated droplet by allowing the retention of specific components and excluding others [71,76]. One example comes from studies of FUS hydrogels [76,77]. FUS hydrogels formed *in vitro* can retain other IDR-containing proteins, like hnRNP A1 and TIA-1, with different avidity, suggesting preferences for some partners over others [76]. Hydrogels assembled *in vitro* also exhibit RNA sequence selectivity. Here, the specificity of binding depends on the RNA-binding modules. For example, FUS hydrogels selectively retain the RNAs that are typically contained in RNP granules where FUS is found *in vivo* [77]. These experiments illustrate how the composition of condensates can result from a collective network of homotypic and heterotypic multivalent interactions between condensate components [63]. Furthermore, the regulation of gene expression influences RNA–protein interactions by modulating the availability, abundance and quality of RNA and RBPs [27,31,47,72].

RNA also contributes to the identity of RNP granules. RNA possesses structural and chemical features, such as multivalency and flexibility, that make it a key component and driver of these compartments [28,78]. The *Ashbya gossypii* RBP Whi3 contains a glutamine-rich IDR that drives the formation of Whi3 condensates *in vivo* and *in vitro* [79]. In this multinucleate fungus, Whi3 forms distinct types of granules that differ by the mRNA species they recruit [80,81]. The interaction of Whi3 with target RNAs changes the conformational ensemble distribution of the RNAs facilitating RNA self-association. These structural rearrangements exclude the possibility of interactions with other non-compatible RNA species by masking critical sequences and therefore contribute to the initiation and the maintenance of specific condensates [82]. The physical properties of Whi3 droplets formed *in vitro*, such as viscosity and their propensity to fuse, depend on the RNA species they contain [79]. These differences may promote the spatial distinction of condensates containing different RNA species and prevent their fusion *in vivo* [79,82]. A spatial distinction is also observed within RNP granules. For example, *Drosophila* germ granules contain several species of RNA that self-assemble in distinct clusters. Because this homotypic self-assembly is sequence-independent, it has been proposed that it arises as a result of the combination of several properties such as RNA length, modifications and structures, and interacting proteins [83]. These examples illustrate how RNA, in addition to its role as a target, can link molecules together participating in the formation of higher-order assemblies with properties distinct from the individual subunits. Overall, the identity of an RNP granule is governed by combinations of specific and unspecific interactions between RNA and proteins [6,28].

## 7. RNA-binding proteins can alter their activity by assembling amyloid-like structures

The ability of some RBPs to assemble into an amyloid-like structure allows these proteins to rapidly transition from a monomeric to a stable assembled state. This switch-like transition is an effective strategy for signal propagation and perpetuation [67,84]. Physical and chemical agents, stressors [67,85], mutations [10,13] or alterations of protein concentration [86] that perturb the IDRs' conformational ensemble may favour the switch to an amyloid or amyloid-like state [13,66]. The ability of RBPs to assemble into amyloid-like structures usually depends on the presence of IDRs with low complexity sequences enriched in asparagine and glutamine, and depleted of β-sheet breakers such as proline and charged residues [56,87,88]. The transition from a monomeric to an amyloid-like state usually alters the biological activity of a protein [66,67]. For RBPs, this can result in changes in their RNA-binding abilities. The formation of fibrils increases the concentration of a protein within a small volume, thereby promoting binding avidity or cooperativity. Moreover, fibril formation can also control how connected domains protrude out of the fibril axis. This arrangement may constrain the RNA-binding modules to specific orientations and hence influence the selection of the RNA targets [12,89] (figure 3*b*).

The neuronal isoform of cytoplasmic polyadenylation element binding protein CPEB regulates synaptic protein translation [90]. Neuronal CPEB from *Aplysia californica* contains a glutamine-rich IDR that behaves like a prion-like domain in yeast [91,92]. Upon a stimulus inducing long-term memory, CPEB multimerizes [93]. This ability to assemble is conserved in Orb2, the neuronal isoform of CPEB of *Drosophila melanogaster* [94–96]. CPEB specifically binds the cytoplasmic polyadenylation element (CPE), a U-rich sequence found in many 3' UTRs through two tandem RRMs followed by a zinc-binding (ZZ) domain [97]. CPEB affects translation of its target mRNAs by controlling the length of their poly(A) tail. Its effects on translation depend on its assembly state; monomeric CPEB promotes poly(A) tail shortening and represses translation, while assembled CPEB stimulates translation by protecting the poly(A) tail from deadenylation and promoting its elongation. CPEB binds to different proteins based on its assembly state; CG13928 binds monomeric CPEB exclusively and contributes to its repressive function, while CG4612 is able to interact with both the monomers and the assemblies and stimulates translation [94]. The high-resolution cryo-electron microscopy structures of Orb2 fibrils purified from *Drosophila* neurons are surrounded by lower-resolution densities that have been interpreted as the RRMs and the ZZ domains projecting out of the axis [95]. Nuclear magnetic resonance studies of *A. californica* CPEB provide further insight as to how amyloid-like fibrils can serve as axes that coordinate the spatial orientation of RNA-binding modules. The CPEB C-terminus, which contains the RNA-binding modules, maintains flexibility when CPEB assembles into fibrils [89]. These studies support a model where the repetitiveness of amyloid-like fibrils contributes to the binding of target mRNAs and protein cofactors through avidity or cooperativity.

Another example of a functional amyloid-like structure comes from the yeast protein Rim4, which functions as a translational repressor. Starvation triggers Rim4 assembly, which results in translational repression of selected transcripts during meiosis [98–100]. Upon completion of meiosis I, the kinase Ime2, a master regulator of meiotic progression [101], triggers the clearance of Rim4 assemblies by multi-site phosphorylation, with the consequent de-repression of target transcripts [100,102]. The increased local concentration of Rim4 upon assembly likely results in an avid binding that could render Rim4 targets unavailable to ribosomes. Rim4 RNA targets do not appear to be enriched for any common motif, raising the question of how Rim4 represses specific transcripts. One speculation is that Rim4 amyloid-like assemblies may reorganize the spatial topology of Rim4 RRMs to establish productive interactions with RNA features not present in the primary sequence. The examples of CPEB and Rim4 illustrate how the assembly of an amyloid-like structure can behave like a switch that alters the function of an RBP.

## 8. Conclusion

Many decades of research have established that structured RNA-binding modules are critical determinants of RNA–protein interactions. However, their general low sequence specificity and binding affinity require that RBPs combine several of these modules to finely tune their binding abilities. In this modular context, the disordered intra-protein linkers that connect RNA-binding modules affect and control the binding properties of an RBP. Additionally, IDRs promoting protein–protein interactions can generate new combinations of RNA-binding modules by connecting different RBPs. Thus, the assembled state of an RBP can have different functional capabilities compared to its unassembled state. As opposed to mutations in well-structured modules, mutations within an IDR are more tolerated, resulting in the rapid evolution of these sequences [31]. However, some mutations could alter the protein's interactome, both by abolishing old or by creating new interactions that can result in dominant gain-of-function phenotypes. These dominant mutations can have physiological consequences in health and disease if the altered or new interactions of a mutant RBP are phenotypically relevant [10,63]. Therefore, studies that compare the interacting partners of RBPs in their assembled and non-assembled state are critical to better understand the function of RBPs.

## Data accessibility

This article has no additional data.
